# How do less‐expensive nitrogen alternatives affect legume sanctions on rhizobia?

**DOI:** 10.1002/ece3.6718

**Published:** 2020-08-31

**Authors:** Ryoko Oono, Katherine E. Muller, Randy Ho, Andres Jimenez Salinas, Robert Ford Denison

**Affiliations:** ^1^ Department of Ecology, Evolution, and Marine Biology University of California Santa Barbara CA USA; ^2^ Department of Ecology, Evolution, and Behavior University of Minnesota – Twin Cities St. Paul MN USA; ^3^ Department of Chemistry and Biochemistry San Diego State University San Diego CA USA; ^4^ Present address: School of Integrated Sciences Cornell University Ithaca NY USA

**Keywords:** fertilizer addition, *Phaseolus vulgaris*, polyhydroxybutyrate, *Rhizobium etli*

## Abstract

The evolutionary stability of mutualistic interactions involving multiple partners requires “sanctioning”–the ability to influence the fitness of each partner based on its respective contribution. Sanctions must be sensitive to even small differences if even slightly less‐beneficial partners could gain a fitness advantage by diverting resources away from the mutualistic service toward their own reproductive fitness. Here, we test whether legume hosts sanction even mediocre N_2_‐fixing rhizobial strains by influencing either nodule growth (which limits rhizobial cell numbers) or carbon accumulation (polyhydroxybutryate or PHB) per rhizobial cell. We also test whether sanctions depend on the availability of less‐expensive nitrogen alternatives, either as nitrate or coinoculation with a more‐efficient isogenic strain. We found that nitrate eliminated differences in nodule size between the mediocre and more‐efficient strains, suggesting that host sanctions were compromised. However, nitrate additions also decreased PHB accumulation by the mediocre strain, which may eliminate any fitness advantages of diverting resources from N_2_ fixation. Coinoculation with a more‐efficient strain could also compromise host sanctions if reduction in fitness from smaller nodules does not offset the potential fitness gain from greater PHB accumulation that we observed in the mediocre strain. Hence, a host's ability to sanction mediocre strains depends not only on alternative sources of nitrogen but also the relative importance of different components of rhizobial fitness.

## Graphical abstract

A legume plant can influence the natural selection of nitrogen‐fixing symbionts, but the strength and effectiveness of this selection will depend on the availability of alternative sources of nitrogen as well as the host's ability to influence different components of rhizobial fitness.

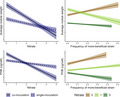

## 1 INTRODUCTION

Human‐induced environmental change is affecting mutualisms that provide significant ecological functions for our ecosystems (Shantz, Lemoine, & Burkepile, 2016; Six, 2009; Kiers, Palmer, Ives, Bruno, & Bronstein, 2010). The evolutionary stability of resource mutualisms could break down in the face of anthropogenic alterations when the relative benefits of traded resources are no longer worth the costs for each partner (Sachs & Simms, 2006). For example, as humans alter soil nutrient supply by applying excess fertilizer, the relative benefits and costs of traded resources to the legume–rhizobia symbiosis are affected. This can potentially alter the exchange rate of benefits (legume C: fixed N) and the strength of selection for mutualistic quality. Evolution of less‐beneficial rhizobial strains could be particularly harmful in agriculture where the ability of N_2_‐fixing rhizobia to meet the nitrogen demands of high‐yield legume crops, like soybean, has been questioned (La Menza, Monzon, Specht, & Grassini, 2017).

In the legume–rhizobia symbiosis, plant hosts preferentially allocate resources and influence relative rhizobial fitness in response to differences in N_2_ fixation among nodules, a process often called “host sanctions.” Host effects on differential resource allocation and relative rhizobial fitness (as measured by differences in nodule size and number of rhizobial cells per nodule, respectively) have been observed widely (e.g., Kiers, Rousseau, West, & Denison, 2003; Simms et al., 2006; Heath & Tiffin, 2009; Oono, Anderson, & Denison, 2011; Regus, Gano, Hollowell, & Sachs, 2014; but see Gubry‐Rangin, Garcia, & Béna, 2010). However, these effects have not been observed as well among nodules that differ only moderately in nitrogen‐fixation rate or efficiency (but see Heath & Tiffin, 2009; Kiers, Rousseau, & Denison, 2006; Quides, Stomackin, Lee, Chang, & Sachs, 2017) nor under alternative environmental conditions, such as nitrate additions (but see Regus et al., 2014; Wendlandt et al., 2019). Even fewer studies have explored how strains that only differ moderately in mutualistic quality are sanctioned under varying environmental conditions (but see Kiers et al., 2006; Regus et al., 2014). And while several studies have concluded that host sanctions remain strong under nitrogen fertilization, these conclusions are typically based on comparisons between one or several effective strains of varying quality and an ineffective strain (Regus et al., 2014; Wendlandt et al., 2019), not between two or more effective strains of varying quality.

If sanctions were not sufficiently severe against moderately less‐beneficial strains that still fix appreciable levels of N_2_, then these strains could divert more resources away from N_2_ fixation to their own reproduction and have higher relative fitness than more‐beneficial strains. This would lead to declining rates of fixation in rhizobial populations. Furthermore, the *severity of sanctions*–the relative fitness between sanctioned and unsanctioned nodules–could depend on environmental conditions, such as fertilizer nitrogen in the soil, to which even legume crops are often exposed because of carry‐over from previous nonfixing crops. When soil nitrogen increases, legumes typically allocate fewer resources toward the symbiosis (Streeter & Wong, 1988; Denison & Harter, 1995; Fujikake et al., 2002; Friel & Friesen, 2019), although there are exceptions among legume species (Regus et al., 2014). But we understand less about the extent to which plants are capable of preferentially allocating resources to reflect the relative performance of each effective nodule on the same plant, sometimes called “relative sanctions” (West, Kiers, Simms, & Denison, 2002), which will play a large role in the evolutionary maintenance of the mutualism in today's changing terrestrial ecosystem.

Models of sanctions by West, Kiers, Pen, and Denison (2002), West, Kiers, Simms, et al. (2002) predict that the availability of soil nitrogen would have negligible effects on the evolution of N_2_ fixation. However, these models assume plants can consistently sanction even moderately less‐beneficial strains when soil N is available, which have not been widely reported. In field studies, while long‐term addition of nitrogen may decrease the relative abundance of beneficial rhizobial genotypes, perhaps by decreasing legume abundance (Weese, Heath, Dentinger, & Lau, 2015), Schmidt, Weese, and Lau (2017) found little effect of crop management on rhizobial mutualism, suggesting that elevated levels of soil nitrogen does not select for poor‐fixing rhizobia. However, crop management treatments alter multiple parameters, such as soil structure and organic content, that could confound the effects of fertilizer on rhizobial evolution. Kiers et al. (2006) also showed that increasing nitrate in the growth media of a soybean cultivar decreased nodule size and rhizobial fitness in both more‐ and less‐beneficial nodules, proportionally. However, in Kiers et al. (2006), the less‐beneficial phenotype was imposed by manipulating N_2_‐gas concentrations around individual nodules. This may not have provided the same fitness benefits for the rhizobia fixing less N_2_ as would strains that fix less N_2_ due to an underlying genetic mechanism. This is because functional nitrogenase still consumes energy (making hydrogen) when nitrogen gas is absent and may prevent manipulated strains from diverting resources to its own reproduction. Hence, in this study, we explored the effects of nitrate on sanctions in a controlled growth chamber using two isogenic strains of rhizobia that vary in mutualistic efficiency due to a genetic basis for a mechanistic link between increased rhizobial fitness and decreased N_2_ fixation.

Mechanistic explanations for differences in efficiency among rhizobial strains are rare. Many less‐efficient strains are simply defective in ways that reduce their own fitness as well as contributions to their host (Friesen, 2012). But, by definition, low‐fitness rhizobial strains will rarely be abundant enough in the field to pose a problem for legumes or the evolution of the mutualism. A bigger threat comes from strains that do increase their own fitness by investing less in their host. For example, a rhizobial cell in a legume root nodule faces a resource‐allocation trade‐off in dividing ATP and reductant between N_2_ fixation and other processes, including synthesis of the lipid polymer, polyhydroxybutyrate (PHB). More PHB can enhance rhizobial survival and reproduction (Muller & Denison, 2018; Ratcliff, Kadam, & Denison, 2008), but Cevallos, Encarnación, Leija, Mora, and Mora (1996) found that a phaC PHB‐negative mutant had prolonged N_2_ fixation and apparently extended the life of the nodules, as indicated by greater plant N on days 38 and 45 and greater final mass on day 59 relative to the wild‐type, PHB(+) *Rhizobium etli*. In preliminary studies, we often observed that nodules containing PHB‐negative strains grew significantly larger than nodules containing wild‐type PHB(+) strains on the same plants, suggesting that the plant can detect and respond to the difference in N_2_ fixation between these strains.

In this study, we explored how a surplus in environmental nitrogen could affect the evolution of a mutualism by measuring the change in a legume host's ability to sanction less‐beneficial, but still effective, strain in the presence of alternative sources of nitrogen. We first confirmed that a PHB‐negative mutant has a greater N_2_‐fixation rate, relative to its respiration cost, than its wild‐type strain. We then assessed the fitness of the “mediocre” wild‐type N_2_‐fixing strain with or without the addition of a less‐expensive source of nitrogen in the form of 1) the PHB‐negative strain, which was more efficient (more N per C respired) and 2) either 1 mM or 5 mM of potassium nitrate. We measured nodule weights as well as PHB accumulation per cell for the wild‐type strain as fitness proxies since these two traits could be inversely related (Hahn & Studer, 1986).

## 2 MATERIALS & METHODS

### 2.1 Plant growth conditions and rhizobial inoculum

*Phaseolus vulgaris* cv. “Royal Burgundy” seeds were surface‐sterilized with 0.09% hypochlorite (3% commercial bleach) for 5 min, rinsed in deionized water multiple times, and incubated in a Petri dish with wet tissue until germination. Rhizobia strains, CE3 (wild‐type, PHB+) or SAM100 (*phaC*; PHB‐negative), were grown in TY media with streptomycin (200 μg/mL) alone or streptomycin (200 μg/ml) plus kanamycin (50 μg/mL), respectively.

To compare fixation efficiencies, germinated seeds were placed in soil microcosms made from two connected Magenta units, which were filled with a 1:1 mixture of vermiculite and sand and then autoclaved. The soil mix was supplied with N‐free nutrient solution (Fujikake et al., 2002) from a reservoir made from a third Magenta unit, via a cotton wick (Figure S1). The nutrient solution was supplemented with 0.5 mM KNO_3_ during the first 14 days after germination to support early plant growth (Laguerre et al., 2012). One to four days after germination, each plant was inoculated with one mL of stationary‐phase inoculum (approx. 10^9^ cells, based on optical density and dilution plating) diluted with 10 ml of starvation buffer (Wei & Bauer, 1998) per plant. Plants were coded so that the randomly assigned strain treatments were not known during efficiency assays. We measured fixation efficiencies for 21 plants (11 for CE3 and 10 for SAM 100) spread across two experimental cohorts in a growth chamber (13 hr day at 25°C, 21°C night). Plants were measured repeatedly between 3 and 10 weeks after sowing to capture developmental changes in N_2_ fixation.

To test host effects on rhizobial fitness when the host has a less‐expensive alternative N source, we used a split‐root method. Germinated seeds were placed in 12.7 cm plastic CYG growth pouches (Mega International, MN) and watered with N‐free nutrient media (Fahraeus, 1957). Plants grew in a Percival growth chamber at 22°C in the dark and at 25°C during the day. The chamber used white and red LED lights that would gradually increase to 75% and 100% capacity (428 µmoles m^−2^ s^−1^ total), respectively, for 8 hr and then decrease for 8 hr to 0%. Pouches were randomly mixed throughout four growth chamber shelves (1.3 m^2^ each) every 3 days. Between 4 and 7 days after germination, the main seedling roots were cut three to four centimeters below the cotyledons to allow lateral root growth into the two halves of the split pouches. Plant root halves were inoculated with rhizobial strains once new roots began to grow into the two halves from the middle. Because the PHB‐negative SAM100 does not nodulate well or as quickly as PHB(+) CE3 (an average delay of 7 days), root halves treated with SAM100 were reinoculated after another week with fresh inoculum. Nitrate treatments were started 3 days after first inoculation.

### 2.2 Nitrogen‐fixation efficiency assay

The N_2_‐fixation efficiency of the two strains was evaluated based on two parameters: the Electron Allocation Coefficient (EAC, fraction of nitrogenase activity making ammonia rather than hydrogen) and the ratio of N_2_ fixation to nodule‐interior respiration. We used Magenta‐box chambers as flow‐through gas‐exchange cuvettes (Oono & Denison, 2010). Hydrogen gas produced by nitrogenase was measured using City Technology 3HYT electrochemical sensors (Witty, 1998). We measured nodulated‐root plus soil respiration as CO_2_ production using Qubit Systems Q‐S151 CO_2_ analyzers. Gas‐flow through each chamber was a mix of O_2_ and either N_2_ or Ar, supplied at 200 ml/min through computer‐controlled Sierra 830L mass‐flow controllers. Gas returning to the H_2_ and CO_2_ sensors was set at 150 ml/min using a Clark MXM‐12 diaphragm pump. Excess flow to the chambers, relative to sampling return flow, prevented influx of the atmosphere into the chambers.

The EAC was calculated as 100% minus the ratio of H_2_ production in N_2_:O_2_ to H_2_ production in Ar:O_2_ (1 − H_2(air)_/H_2(argon)_). We used the peak rate of H_2_ production in Ar:O_2_ because an Ar‐induced decline is commonly observed (Fischinger & Schulze, 2010).

To measure the ratio of the N_2_ fixation to nodule‐interior respiration, we first multiplied the H_2_ production (concentration times supply flow rate) in N_2_:O_2_ mix by the EAC and then by 2/3, based on the relative electron requirements per mole of NH_3_ versus H_2_. To exclude root and soil respiration, we measured the change in N_2_ fixation divided by the change in CO_2_ production with changes in the surrounding O_2_ concentration (in N_2_:O_2_) from 21% to 19% and 17% before returning to 21%. These changes were assumed to affect respiration only in the O_2_‐limited nodule interior, with negligible effects on O_2_‐saturated respiration of root or soil (Oono & Denison, 2010; Witty, Minchin, & Sheehy, 1983). Figure S2a shows a representative assay. Efficiency was then calculated as the slope of a linear regression of N_2_ fixation (calculated from H_2_ production and EAC) on CO_2_ production (Figure S2b).

### 2.3 Less‐expensive N‐alternative experiment

We randomly assigned plants to one of three nitrate treatments: 0 mM, 1 mM, or 5 mM KNO_3_. We chose 5 mM of nitrate as the upper limit because other studies show that 5 mM stops nodule growth in soybeans (Fujikake et al., 2002) whereas nodulation could increase with up to 2 mM (Hussain, Jiang, Broughton, & Gresshoff, 1999). Within each nitrate treatment level, we randomly assigned plants to four inoculation treatments: no inoculation, wild‐type CE3 strain on both root halves, PHB‐negative SAM100 on both root halves, or coinoculation with one strain on each root half. We randomly assigned root halves to one of the two strains for the coinoculation treatments. The coinoculation treatment may approximate field diversity in rhizobial mutualism, even though plants in the field would rarely have as few as two strains. Nitrate treatments are always the same on the two root halves of a plant. We discarded plants early in the experiment whose root halves did not evenly split.

After 7 weeks, we divided individual plants into root halves and shoots for drying and weighing. Final counts for each treatment group after discarding plants due to uneven root splitting or mold growth were 21 control plants (five 0 mM, six 1 mM, ten 5 mM nitrate), 49 wild‐type plants (17 0 mM, 17 1 mM, 15 5 mM nitrate), 47 PHB‐negative plants (17 mM, 15 1 mM, 15 5 mM nitrate), and 60 coinoculated plants (22 0 mM, 19 1 mM, 19 5 mM). Total dried root weights were measured with nodules. Approximately ten random nodules per root half were weighed to assess resource allocation by the plant, with implications for rhizobial fitness. Nodule weight positively correlates with rhizobial cells per nodule, although this linear relationship could differ among strains or nodules with different fixation rates (Oono et al., 2011; Ratcliff, Underbakke, & Denison, 2011). We do not expect this relationship to change across nitrate treatments as long as the strains continued to fix nitrogen at the same efficiency. Nodules were harvested and rinsed with sterile deionized water three times before being crushed in bulk (ten nodules per tube, pooled by plant) in ascorbic acid buffer (Arrese‐Igor, Royuela, & Aparicio‐Tejo, 1992). We stained nodule extracts with Nile red and analyzed rhizobial cells for mean PHB (pg) per cell in the flow cytometer following methods in Ratcliff et al. (2008) on a Guava ExpressPlus. We ran samples with standards whose PHB concentrations had been determined by GC. A conversion equation was developed with the standard samples to calculate the PHB concentration (pg/cell) of the samples. Rhizobial cells were gated with the Guava acquisition software by comparing with a negative control (stained blank sample or unstained cell sample).

### 2.4 Statistics

We compared efficiency measurements between strains using *t* tests on data from two cohorts of plants combining experimental replicates. This test used the means for each plant from repeated measurements between weeks 4 and 9, which excludes early and late developmental stages with low nitrogenase activity. A factorial analysis of variance (Type II ANOVA) was conducted to compare the main effects of inoculum treatments (no inoculation, wild type, coinoculation, PHB‐negative), nitrate treatments (0, 1 mM, and 5 mM), and their interactions on measures of plant and rhizobial fitness (*stats* package of R 3.5.2). We did not analyze the chambers or shelves as random blocks because the plants were randomized regularly among growth chambers and shelves.

## 3 RESULTS

### 3.1 Nitrogen‐fixation efficiency

**FIGURE 1:**
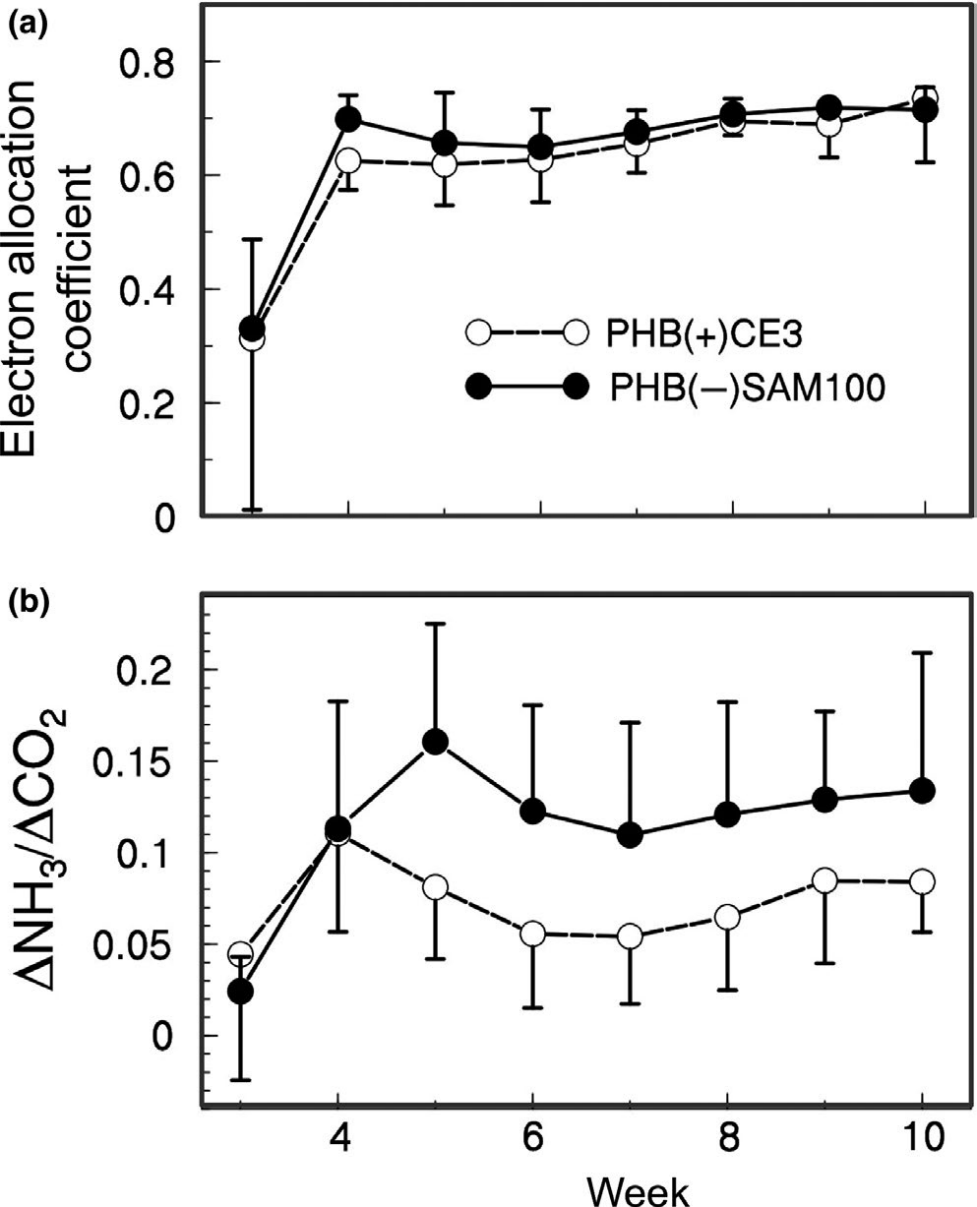
Comparing N_2_‐fixation efficiency between wild‐type and PHB‐negative *Rhizobium etli*. (a) Electron Allocation Coefficient (EAC) of nitrogenase (N_2_ fixation as fraction of total activity, calculated from increase in H_2_ production when switching to N_2_‐free atmosphere) differed little between rhizobial strains. (b) Respiration efficiency of N_2_ fixation (increase in fixation with an increase in respiration, incorporating differences in EAC) was usually greater for the PHB‐negative strain (*t* = −2.48, *df* = 17.8, *p* = .02 for weeks 4–9)

Our efficiency assays confirmed the greater nitrogen‐fixation efficiency of the PHB‐negative strain. The two strains did not differ significantly in Electron Allocation Coefficient (Figure 1a). However, for much of the growth period, the PHB‐negative strain, SAM100, was more efficient than the wild‐type strain, CE3, in the ratio of N_2_ fixation to nodule‐interior respiration (Figure 1b, *t* = −2.48, *df* = 17.8, *p* = .02 for weeks 4–9). Note that our nodule‐interior respiration estimates would include carbon released as CO_2_, but not the additional cost of carbon in PHB granules.

### 3.2 Plant fitness in less‐expensive N‐alternative experiment

**FIGURE 2:**
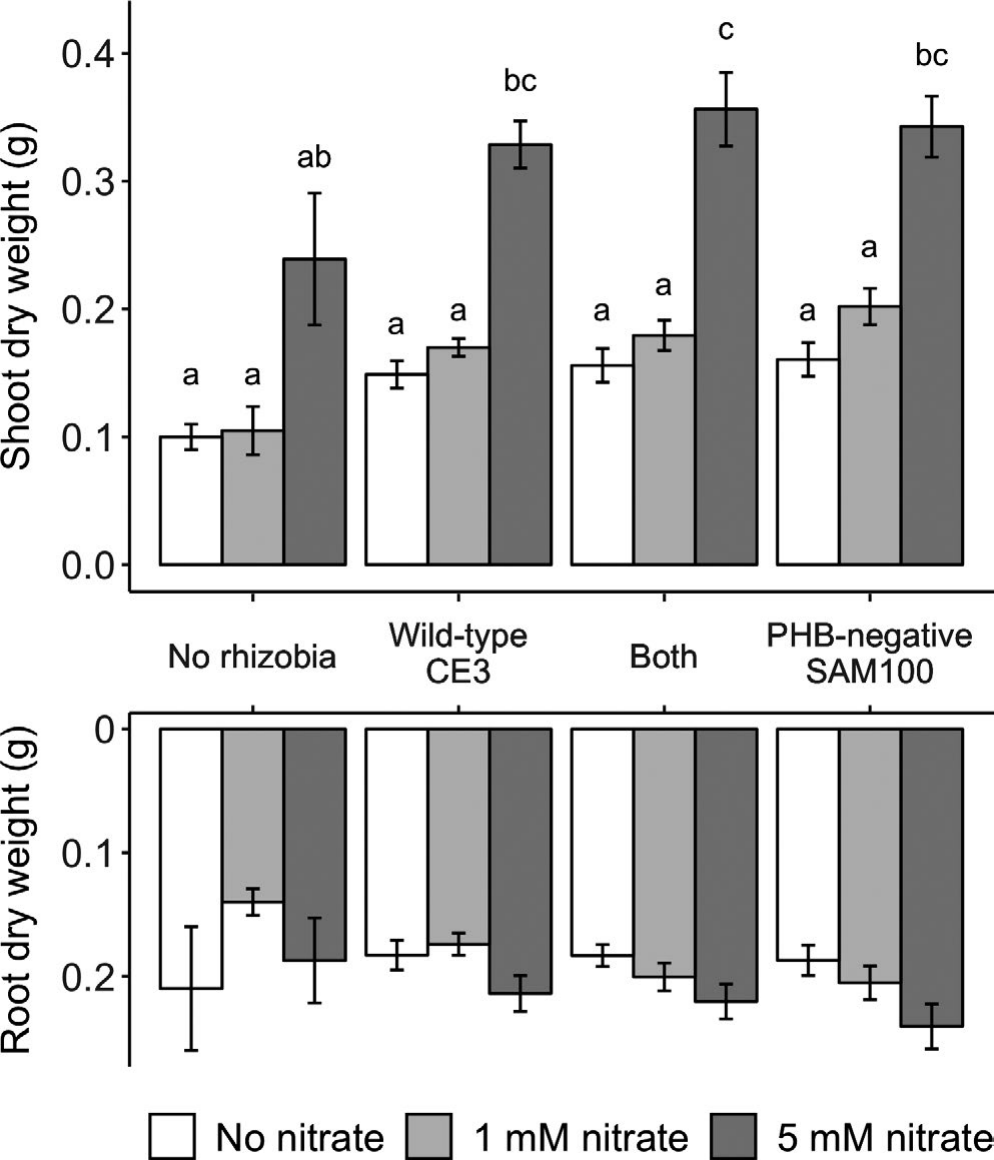
Shoot and root dry weights. Plant shoots increased with nitrate additions and were significantly different across inoculation treatments (*p* < .001). Plant roots also increased with nitrate additions (*p* = .005) and were marginally different across inoculation treatments (*p* = .056). Bars are standard errors. Letters indicate groups with detectable differences based on pairwise post hoc Tukey's HSD comparisons (*p* < .05), which were calculated separately for shoots and roots

**FIGURE 3:**
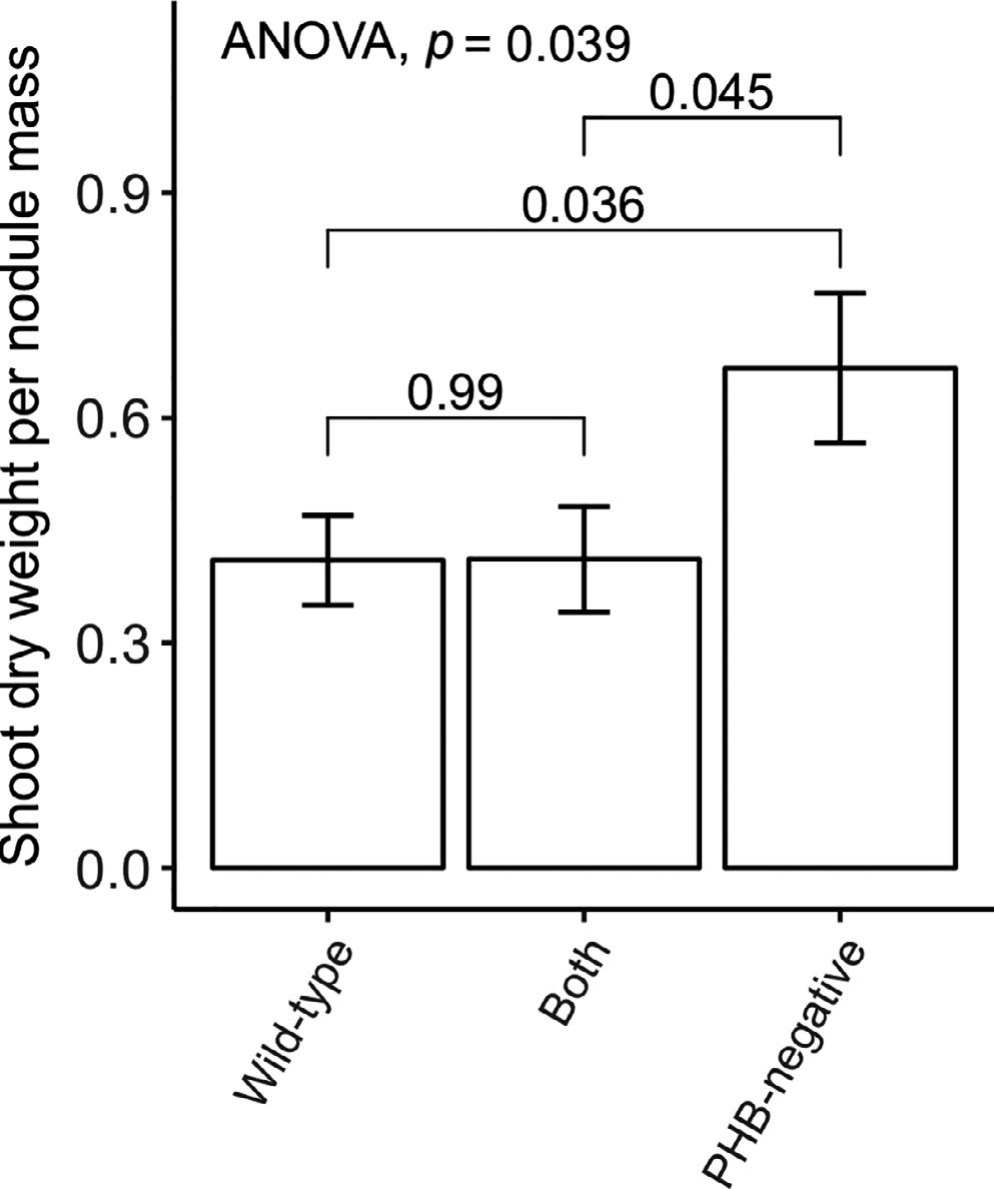
Shoot dry weight per total nodule mass for plants under no nitrate conditions. Plants that were either inoculated by only wild‐type CE3 or coinoculated have lower shoot dry weight per total nodule mass than plants inoculated by only PHB‐negative SAM100 (*p* = .039). Letters indicate groups with detectable differences based on pairwise post hoc Tukey's HSD comparisons (*p* < .05)

We expected plant nitrogen limitation to decrease with either rhizobial inoculation or additional nitrate. However, even inoculated plants were significantly nitrogen‐limited, as shown by large increases in shoot biomass when these plants also received 5 mM nitrate (Figure 2; Table 1; *F*_2,161_ = 79.13, *p* < .001). Rhizobia treatments had smaller effects than expected (*F*_2,161_ = 6.85, *p* < .001), and pairwise post hoc Tukey HSD tests were not always significant. The nitrate treatment also significantly affected the root dry weight (*F*_2,161_ = 5.57, *p* = .005), but post hoc comparisons showed no significant pairwise differences between treatments (Figure 2). Control plants without rhizobial inoculations either did not survive to harvest date and had no nodules (15 out 36), survived with a small number of nodules formed from rhizobial contamination (19 out of 36), or survived with no nodules (2 out of 36). Uninoculated but contaminated plants had significantly fewer nodules 13.1 ± 0.5 *SE* than any of the inoculated treatments (28.5 ± 2.7 *SE* for PHB‐negative or 97.8 ± 5.5 for wild type on coinoculated plants with 5 mM of nitrate). Since all inoculated plants formed nodules and survived to harvest date, contaminated control plants likely make treatment effects conservative. Any nonsignificant comparisons with control plants are mainly due to “survivorship bias” with many non‐nodulating control plants dying or infected by mold before harvest date. The ratio of shoot dry weight to total nodule mass, another measure of rhizobial efficiency, was the greatest for plants that were only inoculated with the PHB‐negative SAM100 strain (*F*_2,53_ = 3.44, *p* = .039). Plants inoculated with wild type had ratios similar to coinoculated plants (Figure 3).

**TABLE 1:** Analysis of variance comparing three nitrate treatments, four inoculation treatments, and their interactions on shoot and root biomass (corresponds to Figure 2)

	*df*	*F*	*p*
Shoot			
*N*	2	79.13	**<.001**
Inoc.	3	6.85	**<.001**
*N* × Inoc.	6	0.29	.94
Residual	161		
Root			
*N*	2	5.57	**.005**
Inoc.	3	2.58	.056
*N* × Inoc.	6	0.69	.656
Residual	161		

### 3.3 Rhizobial fitness in less‐expensive N‐alternative experiment

**FIGURE 4:**
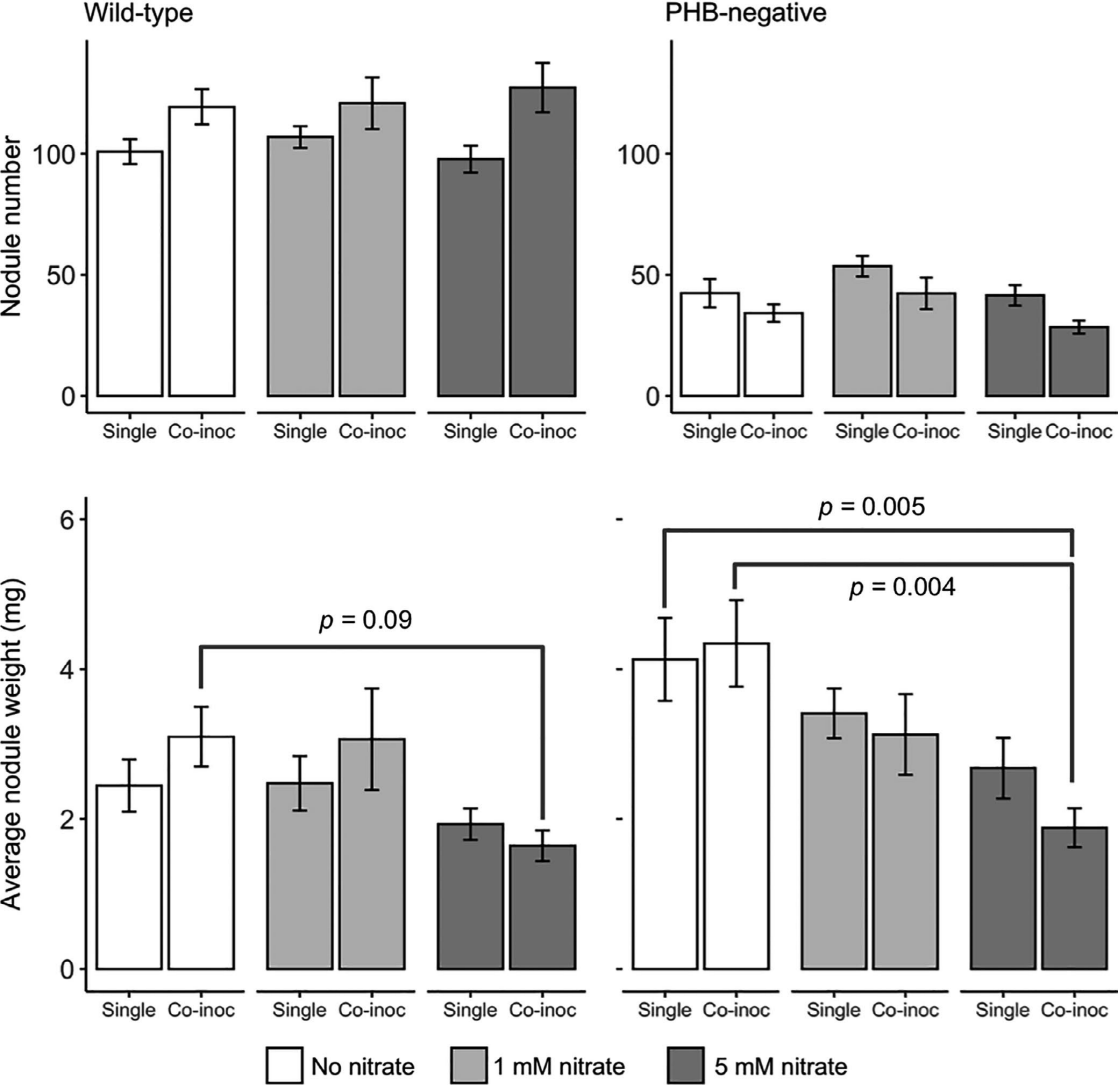
Nodule number and average weight per nodule of each root half. Numbers of nodules on each half root were different between single‐ and coinoculation treatments for both strains. Nitrate affected nodule number more for PHB‐negative SAM100 than for wild‐type CE3. Average weights per nodule decreased significantly with nitrate additions for both strains

We tested how alternative sources of N could affect absolute rhizobial fitness via nodule number, nodule weight, or PHB per rhizobial cell. We found that nitrate levels did not affect nodule number for wild‐type CE3 (*F*_1,103_ = 1.18, *p* = .31) but did for PHB‐negative SAM100 (*F*_2,101_ = 3.58, *p* = .03, Table 2). For both single‐ and coinoculated plants, nodule numbers for the PHB‐negative strain increased marginally from no nitrate to 1 mM nitrate but then decreased with 5 mM nitrate (Figure 4), although none of the three pairwise post hoc tests between N treatments showed significant differences. Wild‐type CE3 made significantly more nodules per root half under coinoculation conditions when the PHB‐negative strain was on the other root half than when they were on both root halves (*F*_1,103_ = 395.00, *p* < .001), whereas PHB‐negative SAM100 had significantly fewer nodules per root half under coinoculation conditions (*F*_1,101_ = 92.78, *p* < .001). The relative frequencies of nodule occupancy by a strain did not significantly change among nitrate treatments (*F*_2,57_ = 1.26, *p* = .29).

**TABLE 2:** Analysis of variance comparing effects of three nitrate treatments, two inoculation treatments (single‐ and coinoculation), and their interactions for wild‐type and PHB‐negative strains separately on number of nodules per root half (corresponds to Figure 4)

Source of variation for nodule no.	*df*	*F*	*p*
Wild‐type CE3			
*N*	2	1.18	.313
Inoc.	1	395.00	**<.001**
*N* × Inoc.	2	0.56	.58
Residual	103		
PHB‐negative SAM100			
*N*	2	3.58	**.031**
Inoc.	1	92.78	**<.001**
*N* × Inoc.	2	0.52	.60
Residual	101		

Increasing nitrate levels consistently decreased average weights per nodule for both strains (*F*_2,103_ = 3.97, *p* = .02 for CE3; *F*_2,101_ = 9.03, *p* < .001 for SAM100, Table 3, Figure 4). The reduction in nodule weight with nitrate addition appeared greater under coinoculation than under single‐inoculations for both strains (Figure 4), although this difference was not statistically significant (Table 3). For example, on coinoculated plants, the addition of 5 mM of nitrate decreased average nodule weights from 3.1 to 1.6 mg for the wild‐type (*p* = .09) and from 4.3 to 1.9 mg for the PHB‐negative strain (*p = *.004). On singly inoculated plants, the addition of 5 mM of nitrate only decreased average nodule weights from 2.5 to 1.9 mg for the wild‐type and from 4.1 to 2.7 mg for the PHB‐negative strain. Post hoc test also showed that the SAM100 nodules under 5 mM of nitrate on coinoculated plants had lower average weight per nodule than when they were singly inoculated on plants without any nitrate (*p* = .005).

**TABLE 3:** Analysis of variance comparing effects of three nitrate treatments, two inoculation treatments (single‐ and coinoculation), and their interactions for wild‐type and PHB‐negative strains separately on average weight per nodule (corresponds to Figure 4)

Source of variation for average wt per nodule	*df*	*F*	*p*
Wild‐type CE3			
*N*	2	3.967	**.022**
Inoc.	1	0.979	.325
*N* × Inoc.	2	0.768	.467
Residual	103		
PHB‐negative SAM100			
*N*	2	9.026	**<.001**
Inoc.	1	1.383	.242
*N* × Inoc.	2	0.338	.713
Residual	101		

**FIGURE 5:**
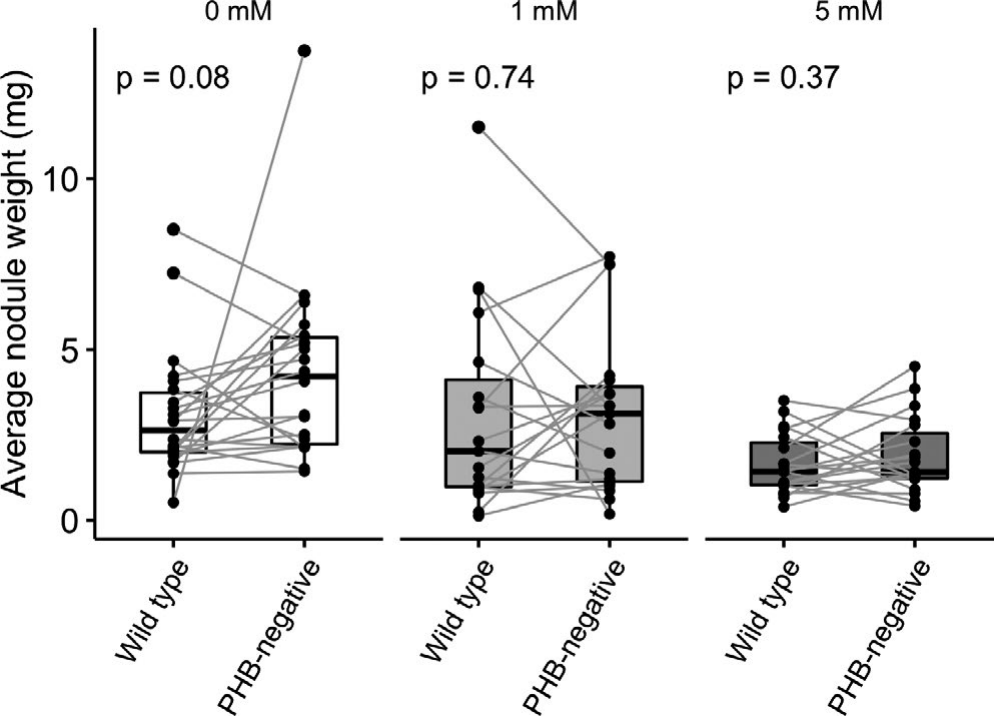
Pairwise comparisons of average nodule weights on each root half on coinoculated plants. Wild‐type CE3 (PHB+) only have marginally lower average nodule weight than PHB‐negative SAM100 under coinoculation conditions with no nitrate. *p‐*values are based on Wilcoxon paired tests

On coinoculated plants, pairwise comparisons between root halves with no nitrate additions showed that the PHB‐negative SAM100 formed marginally larger nodules than the less‐efficient wild‐type CE3 on the opposite side of the same plant (Figure 5; *p = *.08). However, no differences between the two root halves were detected for average nodule weights when 1 mM or 5 mM of nitrate were added (*p* = .74 and 0.34, respectively). The relative frequencies of nodule occupancy by a strain did not affect nodule sizes (Figure S3).

**FIGURE 6:**
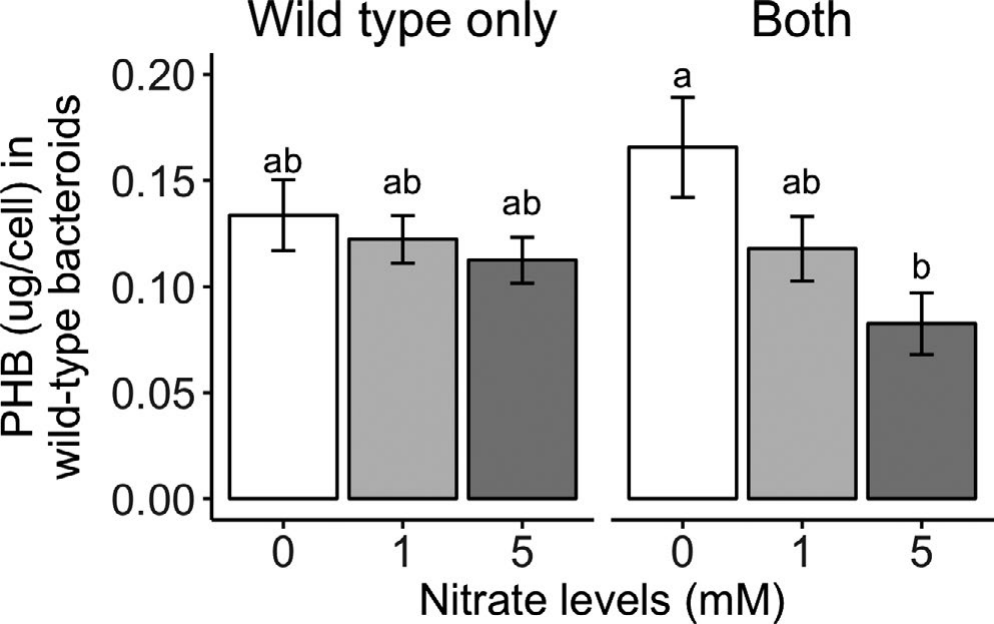
PHB concentrations per cell of wild‐type CE3 under single and coinoculation conditions. Nitrate significantly decreases PHB concentration. The PHB‐negative strain was not analyzed due to low PHB detection with flow cytometry. Letters indicate groups with detectable differences based on pairwise post hoc Tukey's HSD comparisons (*p* < .05)

**FIGURE 7:**
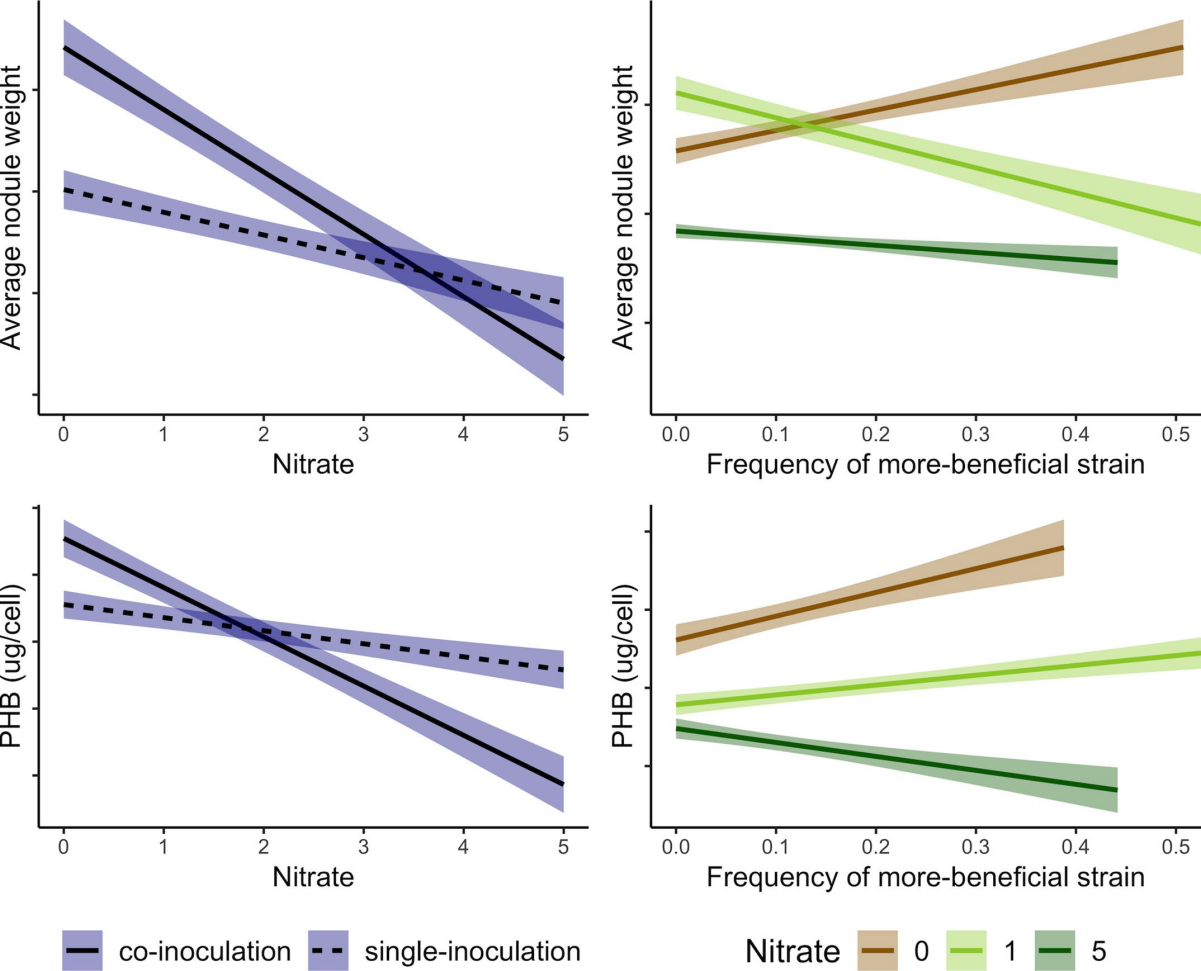
Hypothesized summary of rhizobial fitness trends as a function of two less‐expensive nitrogen alternatives–external nitrate or more‐beneficial strain on the same host. Increasing nitrate decreases absolute rhizobial fitness. The absolute fitness of mediocre strains sharing the same plant with a more‐beneficial strain (coinoculation) will decrease more with nitrate additions than mediocre strains under single‐inoculation. However, the absolute fitness of mediocre strains may increase or decrease as the frequency of more‐beneficial strains increase. Ribbon around regression line denotes 25% confidence interval

Lastly, nitrate significantly decreased PHB accumulation in wild‐type CE3 cells (Figure 6, Table 4). A small subsample of PHB‐negative nodules was analyzed, but no significant PHB‐fluorescence signals could be distinguished from negative controls (unstained cells), as expected. Interestingly, similar to trends in average nodule weights, the reduction in PHB with nitrate addition appeared greater under coinoculation than under single‐inoculation treatments, although this difference was not statistically significant (Figure 6 and prior results not published). On coinoculated plants, the addition of 5 mM of nitrate decreased PHB from 0.17 to 0.08 pg per cell (*p* = .019). On singly inoculated plants, the addition of 5 mM of nitrate only decreased PHB from 0.13 to 0.11 pg per cell (*p = *.94). There was also a slight increase in the average PHB per cell from singly to coinoculated plants in no nitrate conditions (from 0.13 to 0.17 pg per cell) and a slight decrease under 5 mM of nitrate (from 0.11 to 0.08 pg per cell). This trend was, again, similar to what we saw for nodule weights from singly to coinoculated plants, where nodule weights increased slightly from 2.5 to 3.1 mg in no nitrate conditions and decreased slightly under 5 mM of nitrate from 1.9 to 1.6 mg. This suggested to us, given similar trends between the two measures of absolute rhizobial fitness, that mediocre strains are better off under coinoculated conditions than alone under no nitrate (Figure 7). With nitrate, however, mediocre strains are better off alone than sharing their host with the more‐efficient strain. This is inconsistent with the relative sanctions hypothesis (West, Kiers, Simms, et al., 2002) where we expect the availability of a more‐efficient strain to always reduce resource allocation to less‐efficient strains.

**TABLE 4:** Analysis of variance comparing effects of three nitrate treatments, two inoculation treatments (single‐ and coinoculation), and their interactions on PHB concentration in the wild‐type strain (corresponds to Figure 6)

CE3 PHB	*df*	*F*	*p*
*N*	2	4.372	**.0165**
Inoc.	1	0.040	.8417
*N* × Inoc.	2	1.695	.1917
Residual	65		

## 4 DISCUSSION

Many studies (e.g., Kiers et al., 2003; Oono et al., 2011; Regus et al., 2014; Simms et al., 2006) conclude that legume hosts differentiate carbon resources toward symbiotic nodules based on individual nodule performance. The greater plant investment toward fixing nodules relative to nonfixing nodules is consistent across environmental conditions even when less‐expensive nitrogen alternatives are available. Hence, ineffective parasitic strains, while they may prevent hosts from reaching maximum yields, pose little to no threat in the evolution of nitrogen fixation. Moderately less‐beneficial strains that still fix appreciable levels of N_2_, on the other hand, can still outcompete more‐beneficial strains and change the evolution of the mutualism. In this study, we tested whether hosts sufficiently limit nodule growth and PHB accumulation of these less‐beneficial, but still effective, strains that trade‐off mutualistic N_2_ fixation for PHB accumulation. Our fixation efficiency assay (Figure 1) and comparisons of plant biomass per nodule mass (Figure 3) showed that the PHB‐negative SAM100 strain provides more nitrogen relative to its carbon cost than its wild‐type parent, CE3, consistent with previous results (Cevallos et al., 1996). Despite this, we did not see large differences in shoot or root biomasses between the two single‐inoculation treatments (Figure 2), possibly due to significantly lower nodulation rates by the more‐efficient PHB‐negative strain on our bean cultivar (Figure 4). Lower nodulation rates may either be an intrinsic trade‐off with PHB synthesis (Willis & Walker, 1998; Aneja, Zachertowska and Charles, 2005; Quelas, Mongiardini, Perez‐Gimenez, Parisi, & Lodeiro, 2013), a random side effect of the PHB‐knockout mutation, or an interactive effect with the host genotype. Either way, this is an example of how single‐inoculation experiments may underestimate the contributions per nodule of more‐efficient but slower‐nodulating strains that contribute less N overall (Kiers, Ratcliff, & Denison, 2013). To avoid conflating nodulation speed with a strain's contribution, measurements of plant fitness as a function of nodulation frequency by two or more strains could be used (Friesen, 2012; Oono, Denison, & Kiers, 2009). Alternatively, as we have done, direct measurements of fixation efficiency based on acetylene or hydrogen production provide results that would not be confounded by nodulation rates.

Unexpectedly, there were no differences in numbers of nodules per plant with different levels of nitrate for either strain under single‐inoculation conditions (Figure 4). Nodulation rates may not have differed in this study due to limited availability of young, nodulation‐susceptible roots in the hydroponic pouches or because the nitrate treatment was started 3 days after inoculation. However, these seem to be unlikely reasons since we still observed changes in nodule numbers between single and coinoculated treatments where there were more nodules per root half for the wild‐type strain and fewer for the PHB‐negative strain in coinoculated plants. This trend is, again, likely due to the PHB‐negative strain being a slower nodulator than the wild type and the plant not reliably favoring more‐beneficial strains during nodulation. Lack of discrimination is often the case for isogenic strains that differ only in fixation ability (Westhoek et al., 2017), further discrediting the more‐optimistic partner‐choice hypothesis. In any case, changing the total number of nodules per plant would not directly affect the relative fitness of strains unless there was also a change in the relative frequencies of nodule occupancy by a strain among nitrate treatments, which there was not. Furthermore, nodulation has been shown to respond differently to nitrate depending on G x G interactions (Heath, Stock, & Stinchcombe, 2010) and can even increase with nitrate in some other legume species (Regus et al., 2014).

As expected, average nodule weights significantly decreased with higher nitrate levels for both strains (Figure 4). Interestingly, this effect was stronger for the more‐efficient PHB‐negative strain than for the mediocre wild‐type strain. Assuming that each strain's fitness increases with its nodule size, this suggests weaker selection against the mediocre strain relative to the more‐beneficial strain when plants were able to access less‐expensive nitrate. Additionally, this effect of reduced nodule weight with nitrate addition was stronger under coinoculation than under single‐inoculations for both strains (Figure 4, Figure 7). This hinted that nitrate and coinoculation with a more‐beneficial strain have interactive effects on the fitness of the mediocre rhizobia.

With coinoculation, which is more representative of within‐plant diversity in the field, the fitness‐reducing effect of sanctions on the less‐efficient strain's nodule size was reduced or abolished with nitrate additions (Figures 4 & 5), suggesting that nitrogen fertilizer could allow less‐efficient strains to displace a population of more‐efficient strains in the field. Again, these interpretations assume that changes in nodule weight are positively correlated with changes in rhizobia per nodule. Even if the specific relations between nodule size and rhizobial cells per nodule were different between strains, our results still suggest that nitrogen fertilizer increases the relative fitness of less‐efficient strains.

If sanctions severity (as reflected in differences in weight per nodule) depended on the relative benefits of a nodule compared to alternative N sources, rather than only on the absolute fixation rate of a nodule, we might have expected coinoculation with the more‐efficient strain to cause a reduction in the nodule weight of the wild‐type strain, relative to single‐inoculation conditions. However, this did not happen except in the presence of 5 mM of nitrate, when nodule weights for the two strains were not statistically significant (Figure 4, Figure 7). One possibility for why we did not see this effect is that bean plants were still severely nitrogen‐limited, perhaps due to slow nodulation or low numbers of the more‐efficient strain. Indeed, *Phaseolus vulgaris* is reported to have one of the poorest capacities for nitrogen fixation among legumes (Isoi & Yoshida, 1991), suggesting that other legume species may have a greater ability to sanction mediocre strains at lower levels of nitrate.

Finally, nitrate additions not only decreased nodule size but also levels of PHB in wild‐type bacteroids. Hence, although the relative fitness of the mediocre wild‐type strain was improved by additional nitrate in terms of nodule weight, their decreased PHB accumulation per cell, which would have otherwise given these strains a fitness advantage over the more‐beneficial strain, may counter this effect. Similar trends in nodule size and PHB per cell strongly suggest to us that mediocre strains are better off sharing their host with the more‐efficient strain than alone under no nitrate conditions but better off alone on its host when soil nitrate was available (Figure 7).

In summary, less‐expensive nitrogen alternatives in the form of more‐efficient rhizobial strains or soil nitrogen have qualitatively different effects on rhizobial fitness of mediocre strains in common‐bean nodules (Figure 7). In terms of relative fitness, it is unclear whether the larger nodules of the PHB‐negative strain outweigh the PHB advantage of the wild‐type strain, under field‐relevant mixed‐inoculation conditions. The absolute fitness of the mediocre wild‐type strain, based on both nodule growth and PHB per cell, always decreased with nitrate, suggesting that plants effectively “raise the bar” and divert resources away from nodules toward direct nitrogen consumption. Coinoculation with a more‐efficient strain, however, can either increase (based on suggestive data) or have no effect on absolute fitness of the mediocre strain under no nitrate conditions and only decrease absolute fitness under high nitrate conditions. Therefore, a less‐expensive source of nitrogen in the form of external nitrate may not necessarily undermine the ability for hosts to sanction. However, a less‐expensive source of nitrogen from a more‐efficient strain does not always lead to greater sanction severity.

Mutualistic partners can cheat in multiple ways, and we find that measuring fitness benefits that could be mechanistically linked to cheating can reveal important aspects of host sanctions that could otherwise go undetected. The mediocre rhizobial strain in this study could “cheat” in at least two ways–accumulating more PHB or reproducing more within a nodule. We found that nutrient enrichment may not undermine the host's ability to sanction a mediocre rhizobial strain because even though the nodule size difference between high and mediocre performers disappeared, the mediocre strain still reaped less benefits of PHB. In the absence of external nitrate, on the other hand, coinoculation with a high performer can increase both nodule growth and PHB per cell of mediocre strains. Although nodules of the wild‐type (PHB+) strain were smaller than that of the high performer (PHB‐), the greater PHB accumulation by the wild type may ultimately cancel out the effects of sanctions via smaller nodules.

Similar compromises between different fitness benefits may occur in other nutrient exchange symbioses, such as coral symbioses and mycorrhizal associations. In coral symbioses, nutrient loading has been shown to promote parasitism by *Symbiodinium* partners that can benefit from either carbon or nitrogen gains (Baker, Freeman, Wong, Fogel, & Knowlton, 2018). Mycorrhizae benefit from the nutrient exchange with plants via carbon allocation, but their fitness could be measured across multiple spatial and temporal scales, from root tips, biomass in the soil, and sporulation (Chagnon & Bainard, 2015). Because there are usually multiple ways to cheat, we must measure partner fitness with multiple proxies across scales with potential trade‐offs to predict long‐term stability of mutualisms.

Finally, this study measures nitrogen fixation and plant biomass as the only beneficial service by rhizobia while symbiotic bacteria are known for their diverse roles in plant immune response and protection against abiotic stress (Dakora, 2003). It is possible that rhizobial strains that are considered inferior nitrogen‐fixers may provide other benefits to the host that we have ignored here, especially in other environmental contexts or host genotypes. It is also conceivable that the ability to maintain strong sanctions against moderately fixing strains regardless of environmental context is a genetic trait that could vary and be bred within legumes. Exploring natural variations in host sanctions severity in wild populations could help reveal genetic mechanisms underlying plant carbon and rhizobial nitrogen feedback to develop crop genotypes with strong sanctions. However, if surplus nitrogen fertilizer continues to be applied to our agricultural landscapes and leak into surrounding ecosystems, we risk undoing millions of years of natural selection by host organisms for the most‐efficient nitrogen‐fixers.

## CONFLICT OF INTEREST

None declared.

## AUTHOR CONTRIBUTIONS

**Ryoko Oono:** Conceptualization (lead); data curation (lead); formal analysis (lead); investigation (supporting); methodology (lead); project administration (lead); resources (equal); supervision (equal); validation (equal); visualization (lead); writing – original draft (lead); writing – review and editing (lead). **Katherine Muller:** Conceptualization (equal); data curation (equal); formal analysis (equal); investigation (equal); methodology (equal); validation (equal); visualization (equal); writing – original draft (equal); writing – review and editing (equal). **Randy Ho:** Data curation (equal). **Andres Jimenez Salinas:** Data curation (lead); formal analysis (lead); funding acquisition (equal); supervision (equal); writing – review and editing (equal). **R. Ford Denison:** Conceptualization (lead); funding acquisition (equal); investigation (equal); methodology (equal); project administration (lead); resources (equal); software (equal); supervision (equal); writing – review and editing (equal).

## Supporting information

Appendix S1

## Data Availability

Raw data and R codes can be found at Dryad: https://doi.org/10.5061/dryad.xwdbrv19x.
