# Supplementary material for: How do less‐expensive nitrogen alternatives affect legume sanctions on rhizobia?
Source: Ecol Evol. 2020 Aug 31;10(19):10645–56. doi: 10.1002/ece3.6718 (PMC7548176; doi:10.1002/ece3.6718)
Supplement: Supplementary file 1 — Appendix S1 [file ECE3-10-10645-s001.pdf]

**SUPPLEMENTARY FIGURES**

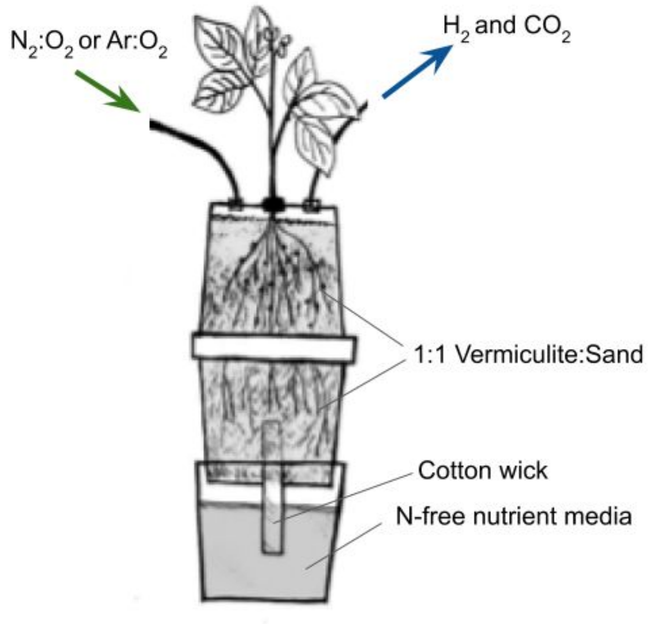


**Suppl. Fig. 1. Chamber used for gas-exchange measurements of nitrogen-fixation efficiency.** Arrows indicate inputs from a gas mixer ($O_2$ mixed with $N_2$ or Ar) and outputs to $H_2$ and $CO_2$ sensors.

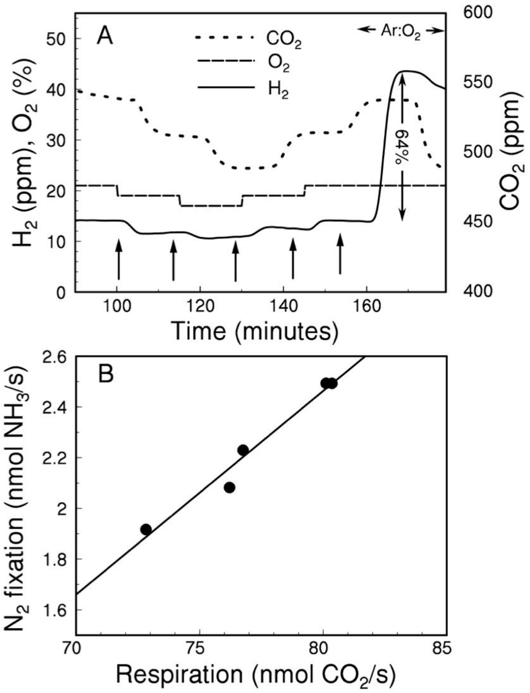


**Suppl. Fig. 2 Measuring nitrogen fixation efficiency by hydrogen gas production across oxygen gradients.** A) Nodule respiration and production of $H_2$ by nitrogenase (which is also fixing $N_2$, when available) respond to changing $O_2$. Switching to a $N_2$-free ($Ar:O_2$) atmosphere directs 100% of nitrogenase activity to $H_2$. B) Nitrogen-fixation rate increases with nodulated-root respiration rate (both calculated from product of concentrations above and flow rate), with slope as one measure of efficiency.

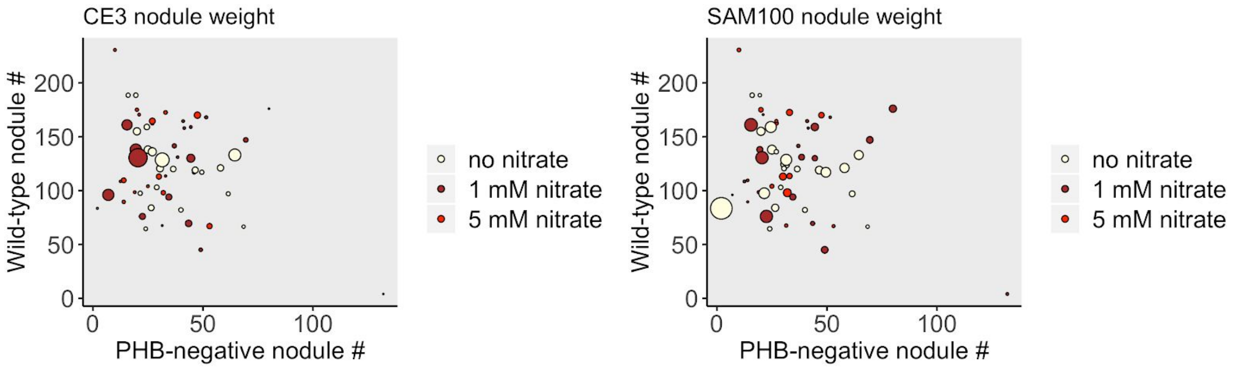


**Suppl. Fig. 3. Correlation between nodule numbers of PHB-negative (x-axis) and wild-type PHB+ (y-axis) strains on co-inoculated plants. Size of points indicate average weight per nodule of wild-type CE3 (left) or PHB-negative SAM100 (right).** No indication that average weight per nodule was correlated with relative nodule numbers of the two strains.
